# Partial volume effect in SPECT & PET imaging and impact on radionuclide dosimetry estimates

**DOI:** 10.22038/AOJNMB.2022.63827.1448

**Published:** 2023

**Authors:** H Marquis, KP Willowson, DL Bailey

**Affiliations:** 1 Sydney Vital Translational Cancer Research Centre, Sydney, Australia; 2 Institute of Medical Physics, University of Sydney, Sydney, Australia; 3 Department of Nuclear Medicine, Royal North Shore Hospital, Sydney, Australia

**Keywords:** PET, SPECT, Partial Volume Effect Radionuclide Dosimetry

## Abstract

**Objective(s):**

The spatial resolution of emission tomographic imaging systems can lead to a significant underestimation in the apparent radioactivity concentration in objects of size comparable to the resolution volume of the system. The aim of this study was to investigate the impact of the partial volume effect (PVE) on clinical imaging in PET and SPECT with current state-of-the-art instrumentation and the implications that this has for radionuclide dosimetry estimates.

**Methods:**

Using the IEC Image Quality Phantom we have measured the underestimation in observed uptake in objects of various sizes for both PET and SPECT imaging conditions. Both single pixel measures (i.e., SUV_max_) and region of interest mean values were examined over a range of object sizes. We have further examined the impact of the PVE on dosimetry estimates in OLINDA in ^177^Lu SPECT imaging based on a subject with multiple somatostatin receptor positive paragangliomas in the head and neck.

**Results:**

In PET, single pixel estimates of uptake are affected for objects less than approximately 18 mm in minor axis with existing systems. In SPECT imaging with medium energy collimators (e.g., for ^177^Lu imaging), however, the underestimates are far greater, where single pixel estimates in objects less than 2-3×the resolution volume are significantly impacted. In SPECT, region of interest mean values are underestimated in objects less than 10 cm in diameter. In the clinical case example, the dosimetry measured with SPECT ranged from more than 60% underestimate in the largest lesion (28×22 mm in maximal cross-section; 10.2 cc volume) to >99% underestimate in the smallest lesion (4×5 mm; 0.06 cc).

**Conclusion:**

The partial volume effect remains a significant factor when estimating radionuclide uptake in vivo, especially in small volumes. Accurate estimates of absorbed dose from radionuclide therapy will be particularly challenging until robust solutions to correct for the PVE are found.

## Introduction

There has been a recent surge in interest in expanding the role of radionuclide therapy in clinical oncology. New peptides, antibodies and radionuclides promise new possibilities in terms of managing metastatic disease. 

 However, today, most treatment regimes follow a “one size fits all” approach when prescribing the amount of therapeutic radiopharmaceutical required (e.g., (1)). The introduction over the last decade and a half of the “theranostic” approach to managing cancer, i.e., combining pre-treatment imaging and therapy using the same targeting agent but varying the radionuclide, provides a means for implementing a more refined and personalised dosing approach. While this has been discussed at length (2) little progress in implementing this into routine clinical practice has occurred. Estimation of the absorbed dose to organs has been extensively reported (e.g., (3)) but there has been little reported on the dose delivered to lesions and metastatic deposits such as in the recent article by Jackson et al (4). This is, in part, due to the large difference in size between many of the organs of interest for dosimetry estimation and the metastatic foci as the spatial resolution of SPECT imaging with higher energy gamma photon emitting radionuclides (e.g., ^177^Lu, ^67^Cu, ^131^I) used for therapy is of the order of 20 mm FWHM or greater. This poor spatial resolution results in a “blurring” of the object below a certain size relative to resolution volume known as the partial volume effect (PVE) which results in the observed uptake of the therapeutic radiopharmaceutical, upon which the dosimetry estimates are based, being significantly underestimated (5). This has proven to be one of the major restrictions in developing measured dose-response relationships for radionuclide therapy for tissues other than whole organs.

 The aim of this work was to investigate the characteristics of the partial volume effect in greater detail to help to understand the limitations that this imposes. A deeper knowledge of the impact that the partial volume effect has in clinical imaging should help researchers to directly address this limitation and develop approaches to overcome it. Previously described methods, such as those summarised in the review by Erlandsson et al (6), have, to date, largely failed to be employed in routine clinical practice, especially in the setting of widespread metastatic disease and in SPECT imaging of radionuclide therapy distributions in theranostics.

***Origins and Definition of the Partial Volume Effect***

**Figure 1:**
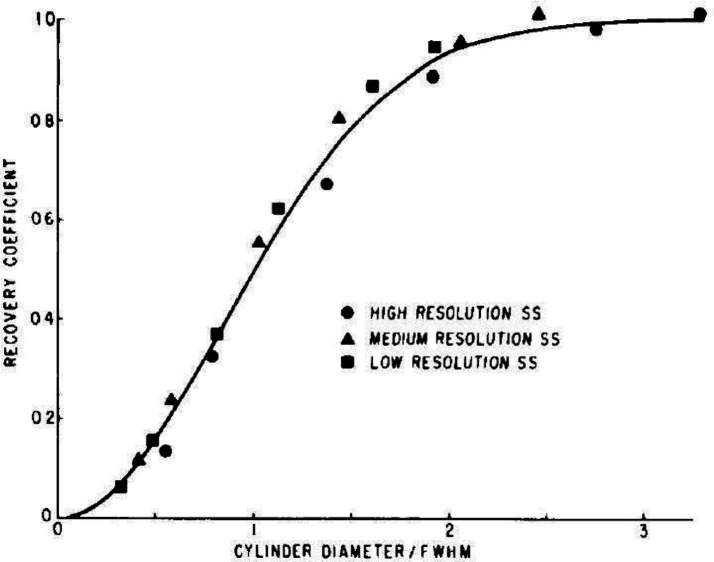
Plot from early report by Hoffman et al on PVE showing the recovery coefficient of radionuclide concentration in cylinders of varying sizes plotted as a function of the system FWHM. The values that have been used to calculate RCs are the peak value in the object. High Resolution – 8.4 mm, Medium Resolution – 12.5 mm, Low Resolution – 18.0 mm. (reproduced with permission)

 The partial volume effect in emission tomography refers to the apparent underestimation of the concentration of the radionuclide in an object in a reconstructed image when the object is of a size that is comparable to some multiple of the measured spatial resolution of the imaging system in terms of its line spread function (LSF) or the Full Width at Half Maximum (FWHM), a measure of the intrinsic “blurring” of the imaging system. In the chapter on Principles and Quantitation in the 1986 book Positron Emission Tomography and Autoradiography (7), Hoffman and Phelps describe the partial volume effect as being “when the object of interest happens to have at least one dimensional smaller than the width of the LSF of the PET system ( 2×FWHM), the object is only partially occupying the sensitive volume of the detectors viewing that dimension. In the reconstructed image this results in an underestimation of the isotope concentration in the structure”. Figure 1 shows the plot of Recovery Coefficient (RC) from the original publication showing the concentration in test objects as a function of object size (8) demonstrating the impact of the partial volume effect. It is worth noting in this work that the authors chose to use the single pixel peak value in the object to determine the recovery coefficient. It can be seen that full recovery in a single maximum pixel is not achieved until approximately 2.5×FWHM.

 In a more recent nuclear medicine physics textbook (9), Cherry et al expands on Figure 1 by showing a cross-sectional profile through cylindrical objects of different diameters and plotting the observed response curve from each (Figure 17-18 and 17-19) in a similar manner to Figure 1. Not explicitly stated, but implied however, is that the recovery coefficient curve shown again represents a single pixel value, representing the maximum voxel value in the region. Such curves are useful when quoting quantitative single pixel biomarkers such as SUV_max_ in PET or SPECT imaging, especially when considering small objects such as lymph nodes or small tumours and the potential underestimation of the radiopharmaceutical concentration observed. To illustrate this with a theoretical example, the above literature suggests in a lymph node measuring 6 mm in its shortest axis measured with FDG PET and surrounded by a relatively low radioactivity background level that the SUV_max_ is likely to be underestimated by some 60-70% on a PET system with FWHM in typical clinical imaging of  7 mm (usually with a post-reconstruction noise-suppressing Gaussian filter applied which contributes to the overall spatial resolution) and will not approach full recovery of the true value until the object measures 18 mm or more. That is, a measured SUV_max_=4, for example, in a 6 mm lymph node is more accurately an SUV_max_ of >10 (=4/(1-0.6)). Further, apparent increases in the SUV_max_ on serial imaging may simply reflect an increase in the size of the lymph node or lesion and not an increase in its metabolic rate, which could be incorrectly interpreted as a poor prognostic indicator.

***Investigating the Impact of the Partial Volume Effect***

**Figure 2:**
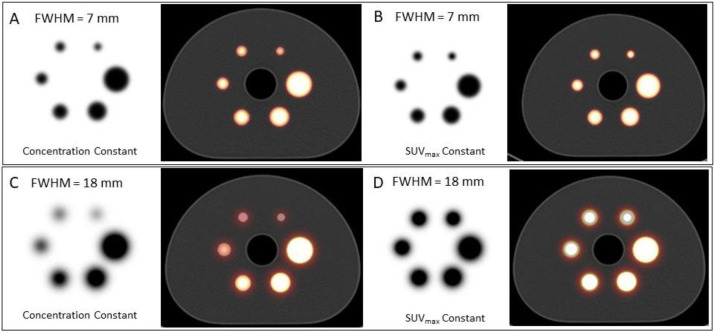
Demonstration of the impact of the partial volume effect for two different spatial resolution values (7 and 18 mm FWHM) in the IEC Body Phantom. The sphere diameters are 10, 13, 17, 22, 28 and 37 mm respectively. On the left in each image is the emission tomographic reconstruction and next to each is the fusion with the CT scan. The two conditions under which the data are presented are for constant concentration in the spheres (**A** & **C**) and for a varying concentration which gives the same reconstructed quantitative value (SUV = 10). In the 18 mm FWHM situation the partial volume effect in image **C** shows a large underestimate in concentration in the three smaller spheres. In image D the large overestimate in the apparent size of the spheres due to the poor spatial resolution is clearly seen

 To explore the impact of the partial volume effect on imaging we have used a finely sampled segmented digital representation of the IEC Body Phantom (with voxel size 1.0×1.0×3.0 mm) and modelled differing concentrations of radioactivity in the spheres of diameters 10, 13, 17, 22, 28 and 37 mm to produce images using clinically appropriate values for spatial resolution in PET (7 mm FWHM) and for medium energy imaging in SPECT (18 mm FWHM) (10). These values are based on measurements from our in-house PET/CT (Biograph mCT/64, Siemens Healthineers, Hoffman Estates, IL, USA) and SPECT/CT (Symbia Intevo.6, Siemens Healthineers, Hoffman Estates, IL, USA) systems. Figure 2 shows the visual impact of the partial volume effect for the two different spatial resolution values under two different conditions: in the first condition the concentration of the radionuclide in the spheres is held constant, while in the second condition the concentration of the radionuclide in the spheres is varied to give a constant reconstructed quantitative response, equivalent to SUV_max_=10.

**Figure 3:**
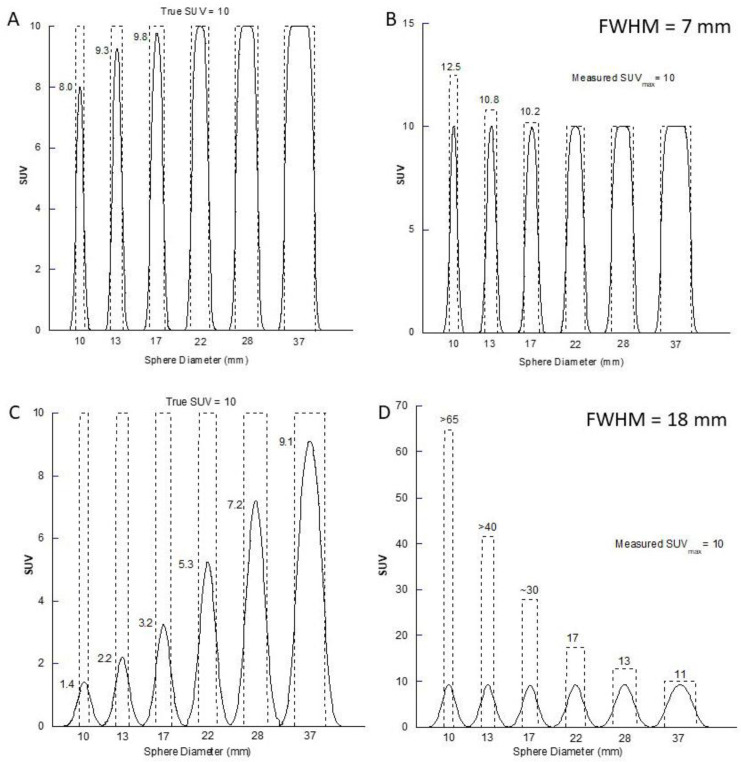
Profiles through the central slice of the six spheres in the IEC Body Phantom for PET- and SPECT-equivalent spatial resolutions of 7 mm (**A** & **B**) and 18 mm (**C** & **D**) FWHM respectively. The sphere diameters vary from 10-37 mm. Graphs **A** & **C** show the conventional recovery profiles for a constant concentration in all spheres equivalent to SUV = 10 (dashed line). Graphs** B** & **D** show the concentration (in SUV units) that would be required to give a measured SUV_max_ = 10 in all spheres

 While the impact for the higher resolution PET situation is relatively minor the effect on the poorer resolution SPECT images is significant. Note in particular the large overestimate in the apparent size of the smaller spheres when the SUV is held constant (Figure 2D). Figure 3 shows profiles through these images for all spheres in the same format (A-D). The profiles in Figure 3A and Figure 3C reflect the recovery values similar to that shown in figure 1 for the two different resolution values. Figures 3B and 3D show the SUV concentrations in the spheres that would be required to give a measured SUV_max_ of 10 in the reconstructed image. 

 Recovery of objects 10 mm and greater is reasonably well performed for the PET resolution. However, for the SPECT resolution of 18 mm the SUV_max_ is still underestimated in the largest sphere ( =37 mm) by approximately 10%, consistent with the Recovery Coefficient curve in Figure 1 for an object diameter approximately 2×FWHM. In graph D a measured SUV_max_ of 10 in the smallest sphere (10 mm diameter) would require a true SUV concentration in the object of >65. It should be noted that the volumes of the smaller spheres in the phantom as measured in the images will not exactly match the true volumes of the physical spheres due to the discretization of the image data.

***Partial Volume Effect and Recovery Coefficients for a Distributed Source***

 While the above examples illustrate important features and limitations of quantitative imaging these only apply to single pixel assessments. With the increasing utility of radionuclide therapy with radiolabelled peptides such as [^177^Lu]Lu-DOTA-Octreotate (“LUTATE”) and [^67^Cu]MecoSAR-Octreotate (“SARTATE”) for treating neuroendocrine tumours, [^177^Lu]Lu-PSMA-617 (“LuPSMA”) for metastatic prostate cancer, interest has turned to the measurement of radiation dose-response from the systemic treatments available. While whole organ absorbed dosimetry estimates are, in general, relatively unaffected by the partial volume effect due to the organ sizes usually being far greater than the spatial resolution value, smaller lesions and areas of abnormality are significantly impacted. As most of the radionuclides used for therapy at present are gamma photon emitters released after β^-^ minus decay they are imaged on the gamma camera with many having gamma photon energies in the medium energy range (180–300 keV). A recent study of the quantitative performance of a state-of-the-art gamma camera when imaging photons of around 200 keV with a standard medium energy collimator demonstrated spatial resolution in the reconstructed images of >20 mm FWHM (10). This would imply from Figure 1 that a single pixel value demonstrating full recovery would not be achieved in masses less than 50+ mm in diameter. This presents a significant challenge for accurate estimation of radiopharmaceutical uptake in tissues of interest, rendering meaningful dosimetry estimates challenging.

**Figure 4:**
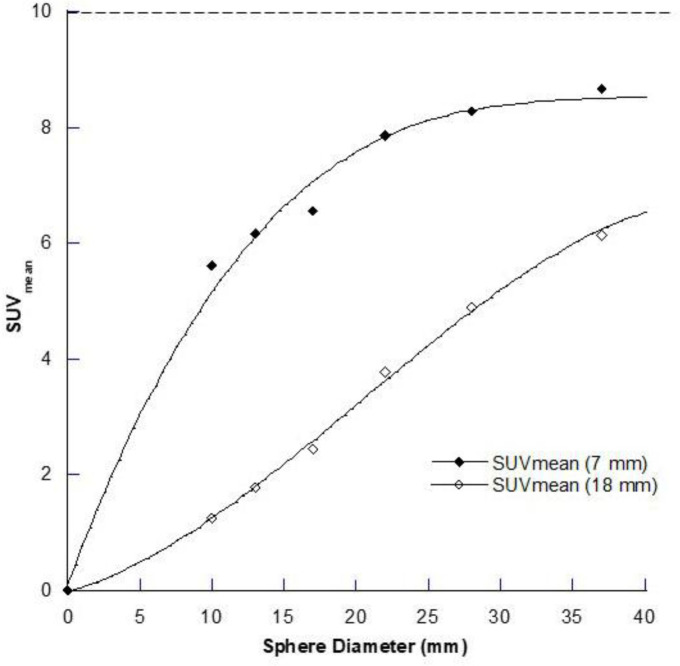
The average apparent concentration (as SUV_mean_) for a ROI on the central slice of the different spheres in the IEC Body Phantom is shown for the 7mm and 18 mm FWHM conditions for a true SUV = 10 (dashed line). The ROI is equal to the size of the sphere

 In addition to this limitation, we are often interested in measuring the average amount of radiopharmaceutical in a volume of tissue similar to what is done with ex vivo gamma counting in preclinical studies where the biodistribution of the radiotracer in different organs and tissues over time is expressed in units of percentage injected dose per gram (%ID/g). Therefore, we are more interested in what the recovery coefficient curve is like for distributed sources in regions or volumes of interest (not in single pixels) equal to the size of the anatomical structure of interest, which can often be identified on morphological imaging when co-registered to the functional PET or SPECT scan. Figure 4 shows the mean value (SUV_mean_) for a region of interest (ROI) through the central slice of the reconstructed image containing the spheres of various diameters, where the region size is set to be the size of the sphere, for the two different spatial resolutions that we have chosen to illustrate the impact of the PVE on quantification. For the 18 mm spatial resolution case, a SUV_mean_ of only  60% of the true value is recovered for objects that measure at least twice the spatial resolution (i.e., 36 mm). The corresponding value for the single pixel SUV_max_ from Figure 1 is nearly 90%.

**Figure 5:**
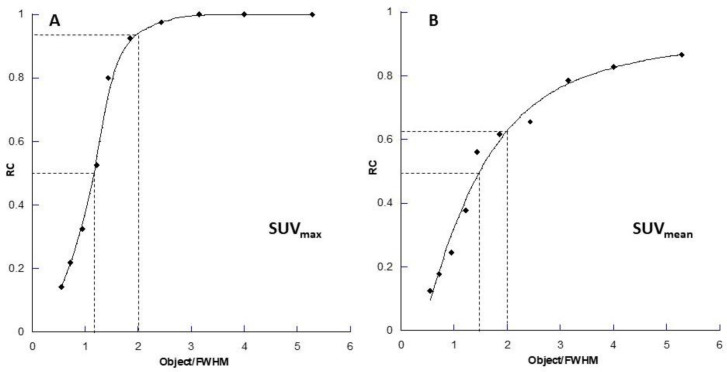
Resolution-independent recovery curves for (**A**) the single pixel (SUV_max_) recovery coefficient (RC) and (**B**) the mean RC within a region of interest equal to the size of the object (SUV_mean_) for the combined 7 mm FWHM and 18 mm FWHM data. The x-axis is plotted as the Object Size/FWHM (as for Fig. 1) so that it is independent of the spatial resolution. Note in the SUV_mean_ recovery curve for a region that occupies the entire “anatomical” space of the object in cross-section full recovery is not obtained even at the maximum ratio of object size to spatial resolution in these data. The dashed lines indicate (i) the 50% recovery point and (ii) the recovery seen at twice the object size relative to the spatial resolution on each graph respectively

Figure 5 shows this as a resolution independent measure: for SUV_max_ 50% recovery occurs when an object is approx. 1.2× the spatial resolution (as defined by the full width at half maximum (FWHM) of a point source profile), whereas for the SUV_mean_ it is at approx. 1.4× FWHM, and for objects of size equal to twice the spatial resolution, the SUV_max_ recovery is over 90% but for SUV_mean_ it is around 60%. The maximum recovery achieved for the SUV_mean_ in a region of interest equal in size to the object is around 90% in objects with size greater than 5× the spatial resolution and full recovery will never be achieved due to the PVE at the edges of the object. For ^177^Lu SPECT imaging this equates to an object of around 90 mm in minimum dimension. In contrast, the SUV_max_ for full recovery is achieved with objects around 45 mm in diameter.

***Example in Radionuclide Dosimetry***

 We present a case example based on contemporaneous MRI, PET and SPECT imaging of a subject with multiple bilateral para-gangliomas in the head and neck adjacent to the carotid vessels who was treated with LUTATE. The PET series was acquired on a Time-of-Flight PET/CT (Biograph mCT/64, Siemens, Hoffman Estates, IL, USA) approximately 1 hour after the intravenous injection of 160 MBq [^68^Ga]DOTA-Octreotate (“DOTATATE”). The subject’s head and neck were scanned for 500 seconds per bed for two bed positions, while the rest of the patient/bed-positions were scanned according to our standard imaging protocol (150 seconds per bed position).

**Table 1:** Dimensions of the four lesions modelled and the uptake measured with PET DOTATATE used in the dosimetry example, based on a subject with somatostatin receptor positive multifocal paragangliomas in the head and neck. The only lesion where full recovery of the SUV_max_ would be expected is in the largest lesion (lesion 1)

**Lesion**	**Size (x) (mm)**	**Size (y) (mm)**	**Size (z) (mm)**	**Volume (cc)**	**SUV**_max_	**Concentration (kBq/cc)**
1	28	22	36	10.2	118	150
2	16	5	15	0.80	58	73
3	6	7	12	0.25	40	50
4	4	5	6	0.06	29	37

 The head and neck data were reconstructed separately from the standard whole-body reconstruction protocol using the vendor’s reconstruction software including CT-based attenuation and scatter correction using the OSEM reconstruction algorithm with 6 iterations of 21 subsets and resolution modelling (RM) enabled with a reconstruction matrix size of 512×512 (with in-plane voxel dimensions of 1.6×1.6 mm, and a z-slice thickness of 3.0 mm). We refer to this reconstruction of the head and neck PET data as a “high-res” PET reconstruction. The high-res PET reconstruction identified four lesions with approximate sizes as shown in table 1.

**Figure 6:**
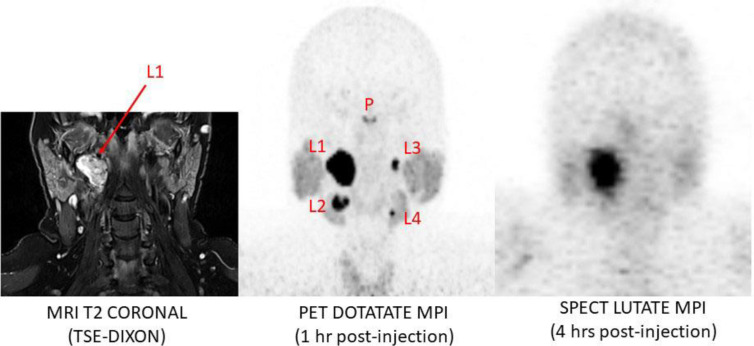
Comparative MRI, PET and SPECT imaging in a subject with somatostatin receptor positive multi-focal paragangliomas of the head and neck. The PET data were used to generate volumes of interest corresponding to the 4 lesions seen (L1 to L4) to examine the effect on dosimetry estimates. Dosimetry estimated by SPECT ranged from a 58% underestimate in the largest lesion (28 × 22 mm in maximal cross-section, 10.2 cc volume) to 99.5% underestimate in the smallest lesion (4 ×5 mm, 0.06 cc). See table 2 for estimates (P = Pituitary gland)

Figure 6 shows a comparison of the MRI, PET and SPECT imaging in this subject. With maximal cross-sectional size of 28×22 mm and based on our results in this study, we would expect the SUV_max_ of the largest lesion imaged with DOTATATE PET/CT to be fully recovered. The lesions yielded measured PET SUV_max_ and radio-concentration values as shown in table 1.

 For this example, based on the results we have reported in this study, we performed the following exercise to examine the impact that the PVE has on SPECT dosimetry estimation. The four lesions on the PET DOTATATE scan were identified in the MRI scan by a radiologist and segmented into Volumes of Interest (VOIs) on the PET scan using an adaptive thresholding technique. Next, the PET measured maximum SUV in the largest lesion, and hence less likely to be affected by the PVE, was determined on the high-res ^68^Ga PET image. This SUV_max_ value (SUV_max_=118) was used to estimate the expected lesion radioactivity concentration for a therapeutic injected dose of 8 GBq of ^177^Lu. This gave a value for the maximum lesion concentration of approximately 9.4 MBq/ml for an injected dose of 8 GBq of LUTATE at 1 hr post-injection. For simplicity and demon-strative purposes, the simulated lesion concentration at 1 hr post injection (not decay corrected) was assigned a nominal concentration of 10 MBq/ml. This activity concentration was assigned to the entire VOI defining each of the lesions and thus it was equal to the mean concentration in all lesions. Of note, there was no background radioactivity in the non-target space and hence no “spill-in” as would occur in a clinical example.

 We estimated the radioactivity remaining in the lesions at 3 later time points (4 hr, 24 hr and 120 hr PI) corresponding to our clinical imaging protocol using an effective half-life with combined physical decay of ^177^Lu and a biological half-life of 55 hrs, based on previously published data (11). OLINDA/EXM dosimetry software and the uniform sphere model was then used with these concentrations to obtain an estimate of the whole lesion dose for each simulated lesion volume using an assumed injection of 8 GBq of LUTATE in a single treatment cycle.

**Table 2:** OLINDA-derived dosimetry estimates in the test case described for the equivalent of an administered therapeutic treatment of 8 GBq ^177^Lu assuming a 55 hour biological half-life in the target tissues

**Lesion**	**Volume (cc)**	**True Dose (Gy)**	**PET-Estimated ** ^177^ **Lu Dose (Gy)**	**Fraction of True Dose**	**SPECT ** ^177^ **Lu Dose (Gy)**	**Fraction of True Dose**
1	10.2	51	37	0.73	19	0.37
2	0.80	50	17	0.34	3.9	0.078
3	0.25	49	10	0.20	1.0	0.020
4	0.06	49	3.6	0.073	0.3	0.0061

 We were, therefore, able to obtain an estimate of the absorbed dose (Gy) in each lesion under the idealised conditions of high spatial resolution with no PVE losses. We then blurred the data with appropriate Gaussian kernels (7 mm FWHM for PET and 18 mm FWHM for SPECT) to replicate the conditions of PET and SPECT imaging. Volumes of Interest for both the PET and SPECT simulated lesions were generated using a 42% threshold to maximum approach (12) and time-activity curves generated for each lesion. These time-activity curves were then input into OLINDA for each VOI where a mono-exponential curve was fit to the data in order to estimate the time-integrated cumulative activity (number of disintegrations, Ã). Next, Ã in each lesion was input into the OLINDA sphere model in order to approximate the absorbed dose to the PET and SPECT simulated lesions. Where a sphere size matching the lesion volume was not available in OLINDA we interpolated between the discrete spheres volumes provided to obtain an estimate of the dose matching the PET-defined tumour volume. In this way we could generate estimates with known “gold standard” values of (a) true absorbed dose to each lesion, (b) PET-estimated ^177^Lu dose based on a notional PET radiotracer with a half-life that could be studied for up to 120 hrs post-injection, and (c) the dose estimate from the ^177^Lu SPECT simulated imaging of the four lesions. The results for the three situations are shown in table 2. It can be seen for all lesions that the PVE causes an underestimation of the true absorbed dose delivered by the treatment based on both PET and SPECT imaging. For the SPECT-derived estimates it ranges from 63% underestimate in the largest lesion to greater than 99% underestimate in the smallest lesion measuring 4×5×6 mm.

## Discussion

***Implications for Quantification and Radionuclide Dosimetry - PET***

 Quantification is heavily relied upon in clinical reports of PET scans, especially with [^18^F]- FDG in cancer imaging. For example, many reports cite higher SUV_max_ values (>8) as being of more concern for a malignant process than lower values (<2.5) (e.g., (13)). The recovery of the single maximum pixel value is affected by a large number of parameters such as the intrinsic spatial resolution of the PET camera, the amount of radiopharmaceutical that is used, the imaging duration, characteristics of the reconstruction algorithm and any post-reconstruction filtering operation, to name but a few. As a consequence, it is hard to compare SUV_max_ values between different PET systems or even identical cameras in different facilities. For this reason, longitudinal studies to monitor response to treatment or similar is best done on the same camera under the same conditions.

 PET enjoys relatively high spatial resolution compared to SPECT. Our PET/CT system (Siemens Biograph mCT/64) has intrinsic spatial resolution of approximately 4.5 mm FWHM, but in the clinical setting, after applying a standard 5 mm FWHM post-reconstruction Gaussian filter to the reconstructed data, the effective spatial resolution in the clinical image is closer to 7.0-7.5 mm FWHM. If resolution recovery is incorporated into the reconstruction process this tends to maintain spatial resolution as a constant across the field of view rather than to improve the overall absolute value. Using this knowledge, we can say that the SUV_max_ in any lymph node or tissue mass less than approximately 18 mm in minimum diameter (on our PET camera) will be underestimated. This can be seen in figure 3A where maximum recovery is achieved somewhere between the 17 mm and 22 mm diameter spheres.

 PET will often demonstrate increased uptake in lymph nodes smaller than what are regarded as radiologically abnormal (<10 mm) but the recovery curves suggest that the SUV_max_ value measured will only represent 0.8 or less of the true value. A situation where this becomes more difficult to interpret is when a lesion such as a lymph node of around this size responds to treatment by shrinking in size, which is likely to be accompanied by a reduction in the SUV_max_ due to the partial volume effect alone. This may be interpreted erroneously, however, as a decrease in the metabolic rate. Thus, even while comparing masses or lesions with interval scanning on the same scanner under as close to identical conditions as possible it may still be misleading to compare the uptake when the volume of the mass or lesion has changed.

***Implications for Quantification and Radionuclide Dosimetry – SPECT & Radionuclide Dosimetry***

 Many of the radionuclides that are now used for therapy emit  ^-^ particles that are used for therapy (e.g., ^177^Lu, ^67^Cu) and gamma photons which can be imaged with SPECT. The spatial resolution of gamma camera-based SPECT systems is dominated by the geometric spatial resolution of the lead collimator. Moreover, the resolution drops off drastically as a function of distance from the collimator to the source. 

 When investigating this effect for ^177^Lu imaging recently we were surprised to find that the reconstructed spatial resolution for our system was >20 mm FWHM (10). With such a large value it is likely that full recovery of radiopharmaceutical uptake will only be achieved in large organs, and not in smaller structures, without further improvements in image processing. Hence, measuring the uptake in cancer tissues from post-treatment SPECT/CT imaging is rarely going to produce results which accurately reflect the true amount of radiopharmaceutical that is in the tissues. 

 This will lead to significant underestimation of the radiation absorbed dose delivered to these tissues making it difficult to establish a true dose-response relationship. We feel that this is one of the major limitations to date in the use of personalised dosimetry for radionuclide therapy.

***Future Directions***

This study of the impact of the partial volume effect in tomographic imaging was borne out of a desire to measure the radiation dose delivered to malignant cancer tissues treated with peptide receptor radionuclide therapy (PRRT) using LUTATE or [^67^Cu]SARTATE (14) imaging with quantitative SPECT (15) in subjects with neuroendocrine tumours that overexpress somatostatin (SSTR2) receptors. We initially measured the radiation absorbed dose in organs such as the kidney using the MIRD-based OLINDA/EXM software package (16) and found that our results were in keeping with other published results in the literature (17). 

 However, when we came to estimating the dosimetry to cancer tissues, which are typically much smaller than whole organs such as the kidney, liver or spleen, we found the values derived to be extremely low and at a level which we could not imagine having any impact on the disease process. Nevertheless, many of the observed metastatic deposits responded to the treatment, which left us perplexed. We now attribute a significant amount of this underestimation in dosimetry to the impact of the limited spatial resolution for medium energy SPECT imaging. Many of the lesions of interest are of a size less than half of the spatial resolution of the gamma camera and therefore are likely to be underestimated by a factor of 5- to 20-fold or more when trying to measure the mean dose to the lesion from a region of interest.

 Radionuclide therapy, unlike chemotherapy, is a systemic treatment that is able to directly demonstrate targeting of the disease process using imaging, which is an enormous potential advantage. The treating team is almost instantly able to form a view of the avidity of the therapeutic for the diseased tissue and therefore the likely outcome of the treatment. However, unlike external beam radiotherapy (EBRT), at present we cannot give an accurate assessment of the amount of radiation delivered to the target. We feel that the poor spatial resolution of SPECT imaging in radionuclide therapy is one of the major impediments to developing a deeper understanding of the relationship between the absorbed dose delivered to tissue and the observed response.

 Numerous strategies have been proposed for decades to mitigate the impact of the partial volume effect. These range from measuring a single pixel or small region of interest and combining the uptake value with the known volume of the tissue to get an estimate of the %ID/cc to sophisticated multimodality imaging techniques which attempt to use a high resolution morphological image to “correct” the blurring due to the poorer spatial resolution of PET or SPECT image (18, 19). None of these have found routine utility in the clinic.

 SPECT reconstruction algorithms incorporating system-specific resolution recovery correction are now standard. These will, in principle, improve the spatial resolution of the reconstructed SPECT image but often at the expense of increasing noise in the reconstructed image. An alternative approach is to use the morphological image from the CT component of the multimodal study to guide the SPECT reconstruction (20). This has been implemented for ^99m^Tc SPECT bone scanning where the skeleton is segmented from the CT scan and is given extra weighting during the reconstruction (21). This is one of the few automated examples where this approach can be used without further image processing as the bones are easy to identify and extract on CT by thresholding based on Hounsfield Units. 

 Improvements in atlas-based tissue classification algorithms are proving useful in identifying normal structures and hence it should be possible to more use an automated approach to identify abnormal tissues. This technique could also be used as a prior to guide the SPECT reconstruction.

 More recently, we have investigated a similar approach using a recently developed “hybrid” reconstruction algorithm known as HKEM (22). This “hybrid kernelised” EM algorithm was designed to use MRI or CT images to guide PET reconstructions. We have taken this further by using a PET image to guide the SPECT image in the theranostic approach, where the same molecule or peptide is imaged with a diagnostic agent (PET) with its higher spatial resolution and then used as side-information to guide the reconstruction of the poorer resolution SPECT image (23).

## Conclusion

 In summary, the teaching and understanding about the partial volume effect has not always emphasised the magnitude of the impact that it has in clinical practice, in particular for distributed sources using regions of interest. 

 SPECT imaging needs to address this issue if it is to make any significant headway towards the routine implementation of personalised dosimetry in radionuclide therapy, the ultimate aim of which is to produce more effective and sustained disease control using radionuclide therapy in subjects with cancer. We may in radionuclide therapy, in fact, already possess Paul Ehrlich’s famous “magic bullet” for cancer treatment but currently we do not know what the most appropriate “calibre” of bullet to use is in any given individual.

## List of Abbreviations

EBRT; External Beam Radiation Therapy

EM; Expectation Maximisation

FWHM; Full-width at half maximum

HKEM; Hybrid Kernelised Expectation Maximisation

LSF; Line Spread Function

MRI; Magnetic Resonance Imaging

OSEM; Ordered Subset Expectation Maximisation

PET; Positron Emission Tomography

PRRT; Peptide Receptor Radionuclide Therapy

PVE; Partial Volume Effect

RC; Recovery Coefficient

RM; Resolution Modelling

ROI; Region of Interest

SPECT; Single Photon Emission Computed Tomography

SUV; Standard Uptake Value

VOI; Volume of Interest
